# Complete mitochondrial genome and phylogenetic analysis of *Mystacoleucus lepturus* (Teleostei, Cypriniformes, Cyprinidae)

**DOI:** 10.1080/23802359.2022.2083993

**Published:** 2022-06-14

**Authors:** Xiao Jiang Chen, Lin Song

**Affiliations:** College of Fisheries Science and Technology, Jiangsu Agri-animal Husbandry Vocational College, Taizhou, PR China

**Keywords:** Mitochondrial genome, *Mystacoleucus lepturus*, phylogeny, Poropuntiinae

## Abstract

In this study, we aimed to sequence and annotate the complete mtDNA genome sequence of *Mystacoleucus lepturus*, which were collected from Luosuojiang River in Menglun area, Yunnan Province, China. The mitochondrial genome was 16,592 bp in length, comprising 13 protein-coding genes (PCGs), 22 transfer RNA genes (tRNAs), two ribosomal RNA genes (rRNAs), and two non-coding regions (origin of L-strand replication and control region). The whole genome contained C (26.5%), A (32.5%), T (25.3%), and G (15.7%), with an obvious A + T bias (57.8%). Based on the concatenated amino acids sequences of 13 PCGs of *M. lepturus* and other 22 fishes, a phylogenetic tree was reconstructed using the maximum-likelihood method. The result of phylogenetic analysis supported a close relationship between *M. lepturus* and *M. marginatus*. The fundamental genetic data of *M. lepturus* would be useful for conservation and phylogeny.

*Mystacoleucus lepturus* (Huang 1979) belongs to the subfamily Poropuntiinae (Cypriniformes, Cyprinidae). It is widely distributed in Yunnan Province of China; Lao People's Democratic Republic and Thailand. *M. lepturus* has most recently been assessed for The IUCN Red List of Threatened Species in 2007, and is listed as vulnerable under criteria A2ce (Jenkins et al. 2009). The typical features that distinguish *M. lepturus* from morphology perspective are dorsal fin rays iv-8; anal fin rays iii-8-9; pectoral fins i-12-15; ventral fin rays ii-8; predorsal scales 8–10; pericaudal handle scale 14; half-moon shaped black brown spots at the base of redorsal scales; black dorsal fin thorn and outer edge of dorsal fin; black narrow edge for inner edge of caudal fin (Wu et al. 1977; Le 2000). Kong et al. (2003) analyzed the *Cytb* and *HVSI* sequences of *M. lepturus* to determine its taxonomic status, and the conclusion was consistent with Huang (1979). This project first determined the complete mitochondrial genome sequence of *M. lepturus*, and a phylogenetic analysis was accomplished with the available mitogenomes sequences among subfamily Poropuntiinae.

Studies involving laboratory animals followed the ARRIVE guidelines (https://arriveguidelines.org/). The specimens of *M. lepturus* (voucher number: ASTIH-21B1108D17) were obtained from Luosuojiang River in Menglun area, Yunnan Province, China (21.660199 N, 101.343714 E) on 15 October 2021. The specimen was preserved in 95% ethanol and stored at Aquatic Science and Technology Institution Herbarium (https://www.jsahvc.edu.cn/, XJ Chen, email: 2007020030@jsahvc.edu.cn). Total genomic DNA was extracted from muscle tissues using Tguide Cell/tissue genomic DNA Extraction Kit (OSR-M401) (Tiangen, Beijing, China) and stored in a deep freezer at −80 °C. The extracted DNA was subjected to sample quality control, DNA library was subsequently constructed and amplified by PCR, followed by size selection and library quality check, finally library pooling and sequencing were carried out on Illumina Hiseq platform 2500 (Genesky Biotechnologies Inc., Shanghai, China). The next-generation sequencing raw data were assembled using MetaSPAdes 3.13.0 (Nurk et al. 2017), and the assembled mitogenome sequences were annotated using the MitoMaker 1.14 (Bernt et al. 2013).

The complete mitogenome of *M. lepturus* was 16,592 bp in length, containing 13 protein-coding genes (PCGs, 3795 bp), 22 transfer RNA genes (tRNAs, 1565), two ribosomal RNA genes (rRNAs, 2,605 bp), and two non-coding regions (an origin of L-strand replication, 32 bp, and a control region, 817 bp). The overall base composition of the *M. lepturus* was C (26.5%), A (32.5%), T (25.3%), and G (15.7%), with an obvious A + T bias (57.8%). The origin of L-strand replication located between *tRNA^Asn^* and *tRNA^Cys^*, and the control region located between *tRNA^Phe^* and *tRNA^Pro^*. Among all 37 genes, nine genes (*tRNA^Gln^*, *tRNA^Ala^*, *tRNA^Asn^*, *tRNA^Cys^*, *tRNA^Tyr^*, *tRNA^Ser(UCN)^*, *tRNA^Glu^*, *tRNA^Pro^*, and *ND6*) were encoded on L-strand, the other 28 genes were encoded on the H-strand. The length of 13 PCGs ranged from 165 bp (*ATP8*) to 1824 bp (*ND5*). Most PCGs initiated with ATG except that *COI* gene initiated with GTG. As for stop codons, seven genes (*ND1*, *COI*, *ATP6*, *COIII*, *ND4L*, *ND5*, and *ND6*) used TAA, three genes (*ND2*, *ATP8*, and *ND3*) used TAG, and three genes (*CO II*, *ND4*, and *Cyt b*) contained an incomplete termination codon (T). The length of tRNA ranged from 67 bp (*tRNA^Cys^*) to 77 bp (*tRNA^Lys^*), while the rRNAs were 952 bp (12*S rRNA*) and 1653 bp (16*S rRNA*) (Table 1).

**Table 1.** Relevant features of *Mystacoleucus lepturus* mitochondrial genome.

	Position					Codon		
Gene	From	To	Nucleotide size (bp)	Amino acid	Space (+) overlap (–)	Initial	Terminal	Strand
*tRNA^Phe^*	1	69	69		0			H
*12SrRNA*	70	1021	952		0			H
*tRNA^Val^*	1024	1095	72		2			H
*16SrRNA*	1118	2770	1653		22			H
*tRNA^Leu(UUR)^*	2788	2863	76		17			H
*ND1*	2864	3838	975	325	0	ATG	TAA	H
*tRNA^Ile^*	3843	3914	72		4			H
*tRNA^Gln^*	3913	3983	71		–2			L
*tRNA^Met^*	3985	4053	69		1			H
*ND2*	4054	5100	1047	348	0	ATG	TAG	H
*tRNA^Trp^*	5099	5169	71		–2			H
*tRNA^Ala^*	5172	5240	69		2			L
*tRNA^Asn^*	5242	5314	73		1			L
OL	5317	5348	32		2			–
*tRNA^Cys^*	5348	5414	67		–1			L
*tRNA^Tyr^*	5414	5484	71		–1			L
*CO I*	5486	7036	1551	516	1	GTG	TAA	H
*tRNA^Ser(UCN)^*	7037	7107	71		0			L
*tRNA^Asp^*	7111	7182	72		3			H
*CO II*	7195	7885	691	230	12	ATG	T	H
*tRNA^Lys^*	7886	7962	77		0			H
*ATPase8*	7964	8128	165	54	1	ATG	TAG	H
*ATPase6*	8122	8805	684	227	–7	ATG	TAA	H
*CO III*	8805	9590	786	261	–1	ATG	TAA	H
*tRNA^Gly^*	9590	9661	72		–1			H
*ND3*	9662	10012	351	116	0	ATG	TAG	H
*tRNA^Arg^*	10011	10080	70		–2			H
*ND4L*	10081	10377	297	98	0	ATG	TAA	H
*ND4*	10371	11751	1381	460	–7	ATG	T	H
*tRNA^His^*	11752	11820	69		0			H
*tRNA^Ser(AGY)^*	11821	11889	69		0			H
*tRNA^Leu(CUN)^*	11891	11963	73		1			H
*ND5*	11967	13790	1824	607	3	ATG	TAA	H
*ND6*	13787	14308	522	173	–4	ATG	TAA	L
*tRNA^Glu^*	14309	14377	69		0			L
*Cyt b*	14383	15523	1141	380	5	ATG	T	H
*tRNA^Thr^*	15524	15596	73		0			H
*tRNA^Pro^*	15596	15665	70		–1			L
Control region	15681	16497	817		0			–

In order to verify the evolutionary relationship, the whole mitochondrial genomes of 18 fish species from nine genera (*Mystacoleucus*, *Sikukia*, *Poropuntius*, *Discherodontus*, *Cyclocheilichthys*, *Cosmochilus*, *Puntioplites*, *Puntius*, and *Sinocyclocheilus*) were selected. Based on the connected amino acids of 13 PCGs, the phylogenetic tree was conducted using the maximum-likelihood method and by MEGA X software (Kumar et al. 2018), and the model (mtREV + G+I) with the lowest Bayesian information criterion (BIC) scores was considered to describe the substitution pattern the best (Jones et al. 1992; Adachi and Hasegawa 1996), with a bootstrap of 1000 replicates. The result of phylogenetic analysis confirmed that *M. lepturus* clustered with *M. marginatus*, and they formed a sister-group with the genus *Sikukia* (*S. gudgeri*), then the above three species formed a sister-group with the genus *Poropuntius* (*P. bantamensis* and *P. normani*). *M. lepturus* was closer to *M. marginatus* (Figure 1). Presently, the studies on *M. lepturus* were limited, and we believed that the fundamental genetic data in this study would be beneficial for further studies on population genetics and evolution of the subfamily Poropuntiinae as well as resource conservation.

**Figure 1.**
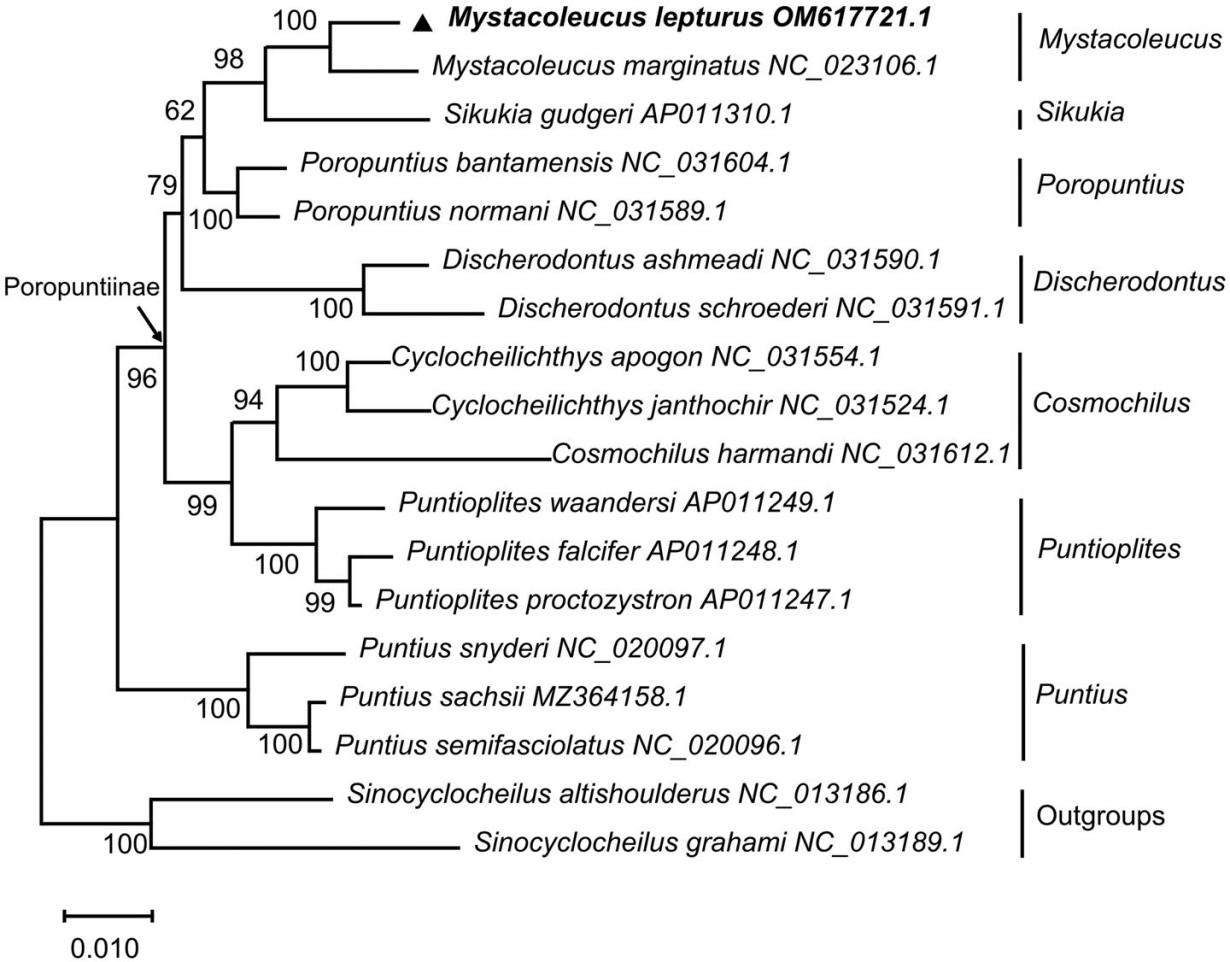
A phylogenetic tree was reconstructed for nine genera (*Mystacoleucus*, *Sikukia*, *Poropuntius*, *Discherodontus*, *Cyclocheilichthys*, *Cosmochilus*, *Puntioplites*, *Puntius*, and *Sinocyclocheilus*), *Sinocyclocheilus* (*S. altishoulderus* and *S. graham*) was used as outgroup, using the maximum-likelihood (ML) method based on the connected aminoacids of 13 PCGs. The tree topology was evaluated by 1000 bootstrap replicates. The species names were followed by their GenBank accession numbers, and the numbers at the nodes represented bootstrap values.

## Ethical approval

Experiments were approved by the Ethical Committee for Animal Experiments of Jiangsu Agri-animal Husbandry Vocational College and conducted following the Chinese Association for the Laboratory Animal Sciences and the Institutional Animal Care and Use Committee (IACUC) protocols.

## Author contributions

Conception and design, XJ Chen and L Song; data curation, XJ Chen; analysis and interpretation of the data, L Song and XJ Chen; funding acquisition, XJ Chen; writing – original draft, XJ Chen, L Song; writing – review and editing, XJ Chen, L Song. All authors agree to be accountable for all aspects of the work in ensuring that questions related to the accuracy or integrity of any part of the work are appropriately investigated and resolved.

## Data Availability

The genome sequence data that support the findings of this study are openly available in GenBank of NCBI at https://www.ncbi.nlm.nih.gov/ under the accession no. OM617721.1. The associated ‘BioProject’, ‘Bio-Sample’, and ‘SRA’ numbers are PRJNA805112, SAMN25829074, and SRR17970748, respectively.
